# Evaluation of Public Engagement Activities to Promote Science in a Zoo Environment

**DOI:** 10.1371/journal.pone.0113395

**Published:** 2014-11-21

**Authors:** Jamie Whitehouse, Bridget M. Waller, Mathilde Chanvin, Emma K. Wallace, Anne M. Schel, Kate Peirce, Heidi Mitchell, Alaina Macri, Katie Slocombe

**Affiliations:** 1 Department of Psychology, University of Portsmouth, Portsmouth, Hampshire, United Kingdom; 2 Department of Psychology, University of York, York, Yorkshire, United Kingdom; 3 Marwell Wildlife, Winchester, Hampshire, United Kingdom; 4 Royal Zoological Society of Scotland, Edinburgh Zoo, Edinburgh, United Kingdom; Universidade de São paulo, Brazil

## Abstract

Scientists are increasing their efforts to promote public engagement with their science, but the efficacy of the methods used is often not scientifically evaluated. Here, we designed, installed and evaluated the educational impact of interactive games on touchscreens at two primate research centres based in zoo environments. The games were designed to promote interest in and understanding of primates and comparative psychology, as a scaffold towards interest in science more generally and with the intention of targeting younger individuals (under 16's). We used systematic observational techniques and questionnaires to assess the impact of the games on zoo visitors. The games facilitated increased interest in psychology and science in zoo visitors, and changed the knowledge of visitors, through demonstration of learning about specific scientific findings nested within the games. The impact of such devices was greatest on younger individuals (under 16's) as they were significantly more likely to engage with the games. On the whole, therefore, this study demonstrates that interactive devices can be successful educational tools, and adds to the growing body of evidence that conducting research on public view in zoos can have a tangible impact on public engagement with science.

## Introduction

Conducting research on public displays at zoos is an effective way to engage with the public about science and can enhance the visitor experience and learning [1], [2]. Installing interactives and other types of signage in these research centres helps to facilitate an understanding about the work being conducted [1] and provide visitors with a more immersive learning environment [3]. Conveying information through interactive devices is becoming more common in zoo settings [4], as the novelty of such devices can help encourage visitors to dwell longer at exhibits [5] and could provide a good addition to how we currently communicate information to the public. Computerised touchscreens, for example, allow the use of dynamic media such as videos and sound which may hold visitor attention more so than static text and imagery [6]. Although many zoos and academic institutions use interactive exhibits to engage their audiences, they are relatively costly and attempts to evaluate the efficacy of such attempts are not as common as they should be [1], [2], [7]. It is critical to demonstrate that these engagement strategies are achieving effective public engagement with science and learning given the considerable financial and time investment in science engagement outside of educational institutions.

Observing how visitors move around exhibits is a common method to assess visitor engagement [1]–[3], [7]. By monitoring visitor ‘dwell times’ for example, we can infer how interested individuals are in particular parts of an exhibit, assuming they would spend more time in areas they are interested in, and less time in areas they are not [2]. Although these methods are useful indicators of the audience's interest, they alone do not allow us to make any assumptions about visitor learning or visitor attitudes. In a study by Waller *et al.* [1], researchers combined observational techniques with questionnaires assessing knowledge, which meant a direct comparison between visitor behaviour and their subsequent learning was possible. Through these methods, the authors demonstrated a greater increase in knowledge in the visitors who interacted with signage, compared to those visitors who did not.

Children behave differently to adults at animal exhibits and respond in different ways to the surrounding information [8]. Ross and Lukas [8] for example, demonstrate that children at an ape exhibit spent less time watching the animals and more time attending to the signs and interpretives than adults. For this reason, it is important to consider the way in which we communicate information when attempting to target young people as a specific audience, through the development of strategies which take advantage of this increased tendency to engage seen in children. Interactive games at zoos may be particularly appropriate to target young people, who often respond well and enthusiastically to computer game based activities [9]. Interactive games therefore, could provide a good medium for increasing interest in science in younger people, and ultimately achieving educational goals.

Here, we assessed the effect of interactive educational games hosted on computerised touchscreens on the interest and learning of zoo visitors at two UK primate research centres: Macaque Study Centre (Marwell Zoo, Winchester) and The Budongo Trail Chimpanzee exhibit (Edinburgh Zoo). The interactive games aimed to increase interest, awareness and understanding of comparative psychology and in particular the relevance of the primate mind and behaviour to understanding the human mind and behaviour, and inspire young people to see science as a potential topic for further study. Quantitative data were obtained through the observation of visitor movements and questionnaire responses.

## Materials and Methods

### Ethics statement

Participation of visitors was entirely voluntary and written informed consent was gained prior to completing the questionnaire. A debriefing sheet was provided after participation. Observational data on dwell times from visitors who did not give informed consent to take part were not used. The procedures were scrutinised and approved by the University of Portsmouth regulated Department of Psychology Ethics Committee and the University of York regulated Department of Psychology Ethics Committee.

### Study Sites

This study was conducted at two zoo-based research facilities for cognitive and behavioural research with primates; The Macaque Study Centre (Marwell Zoo, Winchester, UK; approximately 400,000 visitors per annum) and The Budongo Trail Chimpanzee exhibit (Edinburgh Zoo, Edinburgh, UK; approximately 800,000 visitors per annum). The Macaque Study Centre was designed and built to allow visitors to observe crested macaques (*Macaca nigra, N = 5*) voluntarily participating in cognitive experiments. This facility was built as a collaborative project with the University of Portsmouth, and is used to investigate social cognition and behaviour in macaques, particularly facial expression. Similarly, the Budongo Trail Chimpanzee exhibit at Edinburgh Zoo allows visitors to observe chimpanzees (*Pan troglodytes, N = 18*) voluntarily participating in cognitive experiments. The facility was built by the Royal Zoological Society of Scotland (RZSS), and is run by the Living-Links/Budongo Research Consortium, and investigates chimpanzee social behaviour, communication and cognition. These venues attract a wide visitor demographic with diverse educational backgrounds, and given the large geographical separation of both sites, each should represent separate samples of our target audience.

### Educational Games

**Figure 1:**
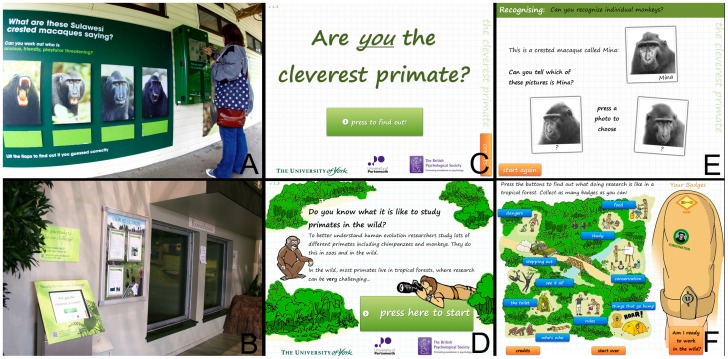
Example of educational games. A) Touchscreen at Marwell Wildlife. B) Touchscreen at Edinburgh Zoo. C) Opening screen of the ‘Cleverest Primate’ game. D) Opening screen of the ‘Research in the Wild’ game. E) Example of ‘Cleverest Primate’ gameplay. F) Example of ‘Research in the Wild’ gameplay.

Two novel interactive games (Cleverest Primate [10], and Research in the Wild [11]) were designed, created and placed on display at both sites, accessible by visitors through a computerised touchscreen (Figure 1). The games alternated (by automation) daily, so only one game was available to play at any one time. As both research centers are so different in layout, and since the aim here was to generalise and not compare the sites, there were no attempts to place devices in comparable locations. This means differences in how the visitors access the touchscreen exist (eg. different distances away from the animals).

The ‘Cleverest Primate’ game was designed to educate the user about comparative psychology. To play the game, the zoo visitor completed different tasks (traditionally and/or recently used by comparative psychologists to explore primate cognition) to understand the socio-cognitive skills primates have, and also to compare their own performance to that of other primates. For instance, visitors were asked if they could match species' vocalizations to the correct referent, identify the individuals from their faces, respond to a human pointing cue and complete a memory task. After each task the visitor was given feedback on their performance and informed or shown (via video) how other primates perform on the same task. At the end the visitor was given a total score and a summary of how their performance compares to other species, in order to understand the pattern of similarities and differences between species.

The ‘Research in the Wild’ game was designed to show users what life is like as a primate researcher in the wild and to think about some of the challenges and benefits of conducting psychological science in a natural environment. Visitors viewed a stylized map of a primate field site, and navigated via various icons to explore different aspects of research in the forest. Clicking/touching each icon lead to a multiple choice quiz question, such as guessing the average time spent walking each day and what a typical dinner would be. Through this they could get a sense of the reality of living in a remote forest (basic food, long drop toilet, night noises) as well as the challenges for collecting data on wild primate behaviour (walking to find and study the primates, identifying the primates, dangers in the forest such as biting ants and snakes, and respecting the safety and wellbeing of the primates). Visitors were awarded up to nine virtual badges for each aspect of life as a primate researcher in the wild that they successfully found out about.

### Observational Procedure

This study ran from 18th February to 30th August 2013 (installation of touchscreen; 31st March at Marwell Wildlife, and 23rd April at Edinburgh Zoo) and involved 1084 participants (586 at Marwell Wildlife, 498 at Edinburgh Zoo). Observations using one/zero sampling [12] were taken to record visitors' activity as they entered and navigated the exhibits at the Macaque Study Centre and the Budongo Trail Chimpanzee exhibit. At Marwell, data were collected on whether the visitors interacted with the touchscreen and games, approached the research window (and if the researcher was currently present) or approached the interactive signage which were already installed at the exhibit. At Edinburgh, data was collected on whether the visitors interacted with the touchscreens and games only, as there was no cognitive research occurring at this time. We defined interaction with the touchscreen as any individual playing the game or observing the game being played. Approaching was defined as pausing within arms reach of the touchscreen and orienting towards it. Dwell times were recorded using a stopwatch. Total time at the touchscreen was recorded at both sites in addition to the total time spent at the exhibit at Marwell and total time spent in the atrium area of the Budongo Trail exhibit, before proceeding upstairs to see the chimpanzees.

When exiting the exhibit (Marwell) or Atrium (Edinburgh), visitors were asked if they wished to participate in the study. If visitors did not want to participate, the observational data collected on their dwell times was erased. Willing visitors signed an informed consent form and became a participant of this study. We administered a questionnaire comprised of a series of questions about their opinions of primate research, understanding of primate behaviour and interest in science and demographic questions about their age, sex, and educational background.

### Questionnaire

Participants were asked to complete an 11-item questionnaire (Table 1. Questions 1–11) relating to their attitudes and understanding of primate research, and about information contained within the games. Responses were recorded on 7-point likert scales, with anchors appropriate to the question being asked (eg. 1, Not at all – 7, Very much). Individuals who interacted with the touchscreen were asked two more questions about their experiences with the games (Table 1. Questions 12–13).

**Table 1:** Quesionaire questions and factor loadings.

	Component					
	1		2		3	
Question	M	E	M	E	M	E
1. Do you think we can learn about how humans think by studying primates? (Not at all – Completely)	.**479**	.219	.071	**.482**	.292	−.312
2. Do you think that primates can tell each other apart from their faces? (Not at all – Very easily)	-					
3. Do you think that primates can understand pointing? (Not at all – Very easily)	-					
4. Which of these should researchers NOT do when studying primates in the wild? (Sneeze Touch Listen Watch Feed)	-					
5. Do you think that most primate species are safe from extinction? (No, not at all – Yes, we can)	.208	−.046	**.743**	−.170	−.275	**.834**
6. Do you think we can stop primates from going extinct if we want to? (No, not at all – Yes, we can)	.157	.154	.038	.549	**.749**	**.577**
7. Do you think we should stop primates from going extinct? (No, not at all – Yes, it is very important)	.047	.057	−.205	.**827**	**.774**	−.018
8. Are you interested in learning more about science? (Not at all – Yes, very much)	**.659**	**.959**	−.518	−.011	.048	.017
9. Are you interested in learning more about psychology? (Not at all – Yes, very much)	**.825**	**.793**	−.175	.021	−.024	−.055
10 Do you think psychology is a scientific subject? (Not at all – Yes, very much)	**.738**	**.539**	.031	.152	.133	.057
11. Do you think science is boring? (Not at all – Yes, very much)	**−.589**	**−.736**	.094	−.038	.519	.069
12. Did you play on the interactive touchscreen? (Yes/Yes, I watched others/No)	-					
13. How much did you enjoy the games? (Not at all – I really enjoyed the games)	-					

Factor loadings. Comparision of the two sites; M = Marwell, E = Edinburgh. Highest factor loadings in bold.

The answers to Question 2–4 could be obtained from one of the interactive games, and were therefore used as an indication of whether scientific understanding increased as a result of having played the relevant game. Questions 5–7 focused on conservation issues, and were used to explore the games affect on participant's opinion of issues relating to primate extinction and conservation. Finally, we asked Questions 8–11 to investigate the effects the games had on visitor interest in the psychological sciences.

### Questionnaire Responses: Principal Components Analysis

The responses given to Questions 1 and 5–11 were subjected to principal components analysis (PCA) to reduce the data into suitable components for further statistical tests [13]. As Questions 2–4 were fact-based and not opinion-based questions (unlike the majority of questions), they were therefore subject to their own analysis. PCA allows for grouping of correlated variables into components, and therefore for data can be analysed based on any important underlying structures [1]. The Kaiser-Meyer-Olkin test (sampling adequacy: 0.67) and the Bartlett's test of sphericity (*X^2^* = 1305.2, *p*<0.001) confirmed suitability of the dataset to PCA. We reduced the data using a varimax rotation into three components (see Table 1: accounting for 30.4%, 15.3% and 13.1% of the variance), allocating questions to components based on the highest factor loadings, whilst ignoring weak factor scores (<0.50). Exploration of the results, however, found only a single stable component between sites (component 1). Component 1 was therefore the only component subjected to further analyses, and was comprised of Questions 8–11; ‘Are you interested in learning more about science?’, ‘Are you interested in learning more about psychology?’, ‘Do you think psychology in a scientific subject?’ and ‘Do you think science is boring?’. These items appeared to relate to (and will be further known as) ‘*Interest in science*’. A reliability analysis of the items in this component suggested they were highly related (Cronbach's α = 0.88). Factor loadings were transformed to a normal distribution (Kolmogorov-smirnov Z = 1.29, *p* = 0.073) and therefore suitable for parametric analysis.

### Design and analysis

Observational and questionnaire data were collected both before (pre-touchscreen condition) and after (touchscreen condition) the installation of the touchscreen. The study employed a quasi-experimental independent groups design, using whether the touchscreen was present, whether the participant played the game and which game was available to play as independent variables. Visitor interest in science, learning about specific issues and understanding of conservation issues were treated as dependent variables.

Scale questionnaire data were reduced using factor analysis to be subjected to parametric statistical analysis. If identified as non-normal, factor scores were transformed (using a Log10 transformation) and so did not deviate from a normal distribution (Kolmogorov-Smirnov test). Data which could not be transformed were subjected to non-parametric tests. Significance level was set at 0.05 for all analyses.

## Results

### Visitor composition

Of the 1084 visitors who agreed to take part in this study, 894 participated after the installation of the touchscreen. More than half of these were female (646/1084) however these differences in male/female composition did not differ between sites (Chi-square test, (*X^2^* = 3.34, *p* = 0.060), and did not differ between participants who had played the game or not (*X^2^* = 1.55, *p* = 0.213). The average age of visitors was 24.9±12.6 yrs, (range 5 yrs–68 yrs), however the mean age of individuals was lower after the installation of the touchscreen (before installation, 30.1±14.5 yrs; after installation 23.9±11.9 yrs; independent samples t-test, *t* (1059) = −6.33, *p* = <0.001). This difference though, is likely explained by the fact the installation of the study coincided with the beginning of UK school summer holidays.

### Touchscreen summary data

To estimate how many visitors interacted with the games, we assessed how many visitors entering Budongo Trail on a test day at Edinburgh Zoo interacted with the game and how many did not. Overall at Edinburgh, 24% (out of 82) of visitors played on the games, but this differed between age groups. Of these, we found that 45% of visitors who played on the games fell within of our target age group (estimated at 16 or less.).

The average time visitors spent playing the touchscreen game was 2.31 minutes, which did not differ significantly between the sites (*t* (358) = 0.74, *p* = 0.461). Visitors spent more time playing the ‘Cleverest primate’ game (mean, 2:48±1:23) than the ‘Research in the wild’ game (Mean = 2:05±1:35), *t* (358) = 4.39, *p* = <0.001). On a scale of 1–7 (not at all – very much) of enjoyment, visitors rated ‘Cleverest primate’ as significantly more enjoyable (5.63±1.3) than ‘Research in the wild’ (4.87±1.7) (Mann-Whitney U: *Z* = −4.01, *p*<0.001). Individuals who interacted with the touchscreen spent, on average, twice as long in the exhibit at Marwell (*t* (384) = 9.82 *p* = <0.001, played the game: 5:01 min±2:45; didn't play the game: 2:39 min±1:52), and twice as long in the atrium at Edinburgh (*t* (495) = 8.33 *p* = 0.036, played the game: 3:35±2.03; didn't play the game: 1:14±3.50).

Of the younger visitors who participated in the study (defined as 16 years old or less), 47.0% interacted with the touchscreen. This was significantly more than the older visitors (35.2%: *X^2^* = 10.29, *p* = 0.006). Young visitors however, were not more likely to engage with the interactive signage or approach the research stations (Marwell wildlife only)(*X^2^* = 0.05, *p* = 0.822 and *X^2^* = 1.23, *p* = 0.541 respectively). The duration of time spend in the exhibits or on the touchscreens did not differ between age groups (*t* (384) = −0.18 *p* = 0.241, and (*t* (1021) = 0.632 *p* = 0.527 respectively).

### Impact of the games on *interest in science*

The questionnaire component *interest in science* was used as a dependent variable in a 2 (played with game) ×2 (game type) between subjects ANOVA. Individuals who did not play the game did not differ in this component to individuals who were sampled before the installation of the touchscreen (*t* (186) = −0.27, *p* = 0.785), so these two groups were combined. No significant relationship with age was found (F = 1.42, *p* = 0.233, η^2^ = 0.002), so age was not used as a covariate in the study. *Interest in science* did not change depending on which of the two games was available to the visitor (F = 0.78, *p* = 0.379, η^2^ = 0.001) or if they played the game (F = 2.06, *p* = 0.151, η^2^ = 0.003). However, a significant positive interaction was found between playing the game and which of the two game types was available (F = 4.84, *p* = 0.028, η^2^ = 0.006). Therefore, if visitors played on the ‘Cleverest Primate’ game, they reported a significantly greater *interest in science*. Games did not have an effect on visitor opinions of conservation issues (Table 1: Q5, Q6, Q7, Z = −.14, *p* = .892, Z = −.26, *p* = .792 and Z = −.718, *p* = .473). Neither did games affect visitors perception of psychology as a scientific subject (Cleverest Primate; Z = −1.22, *p* = .223, Research in the Wild; Z = .77, *p* = .441).

### Impact of games on visitor knowledge

**Figure 2:**
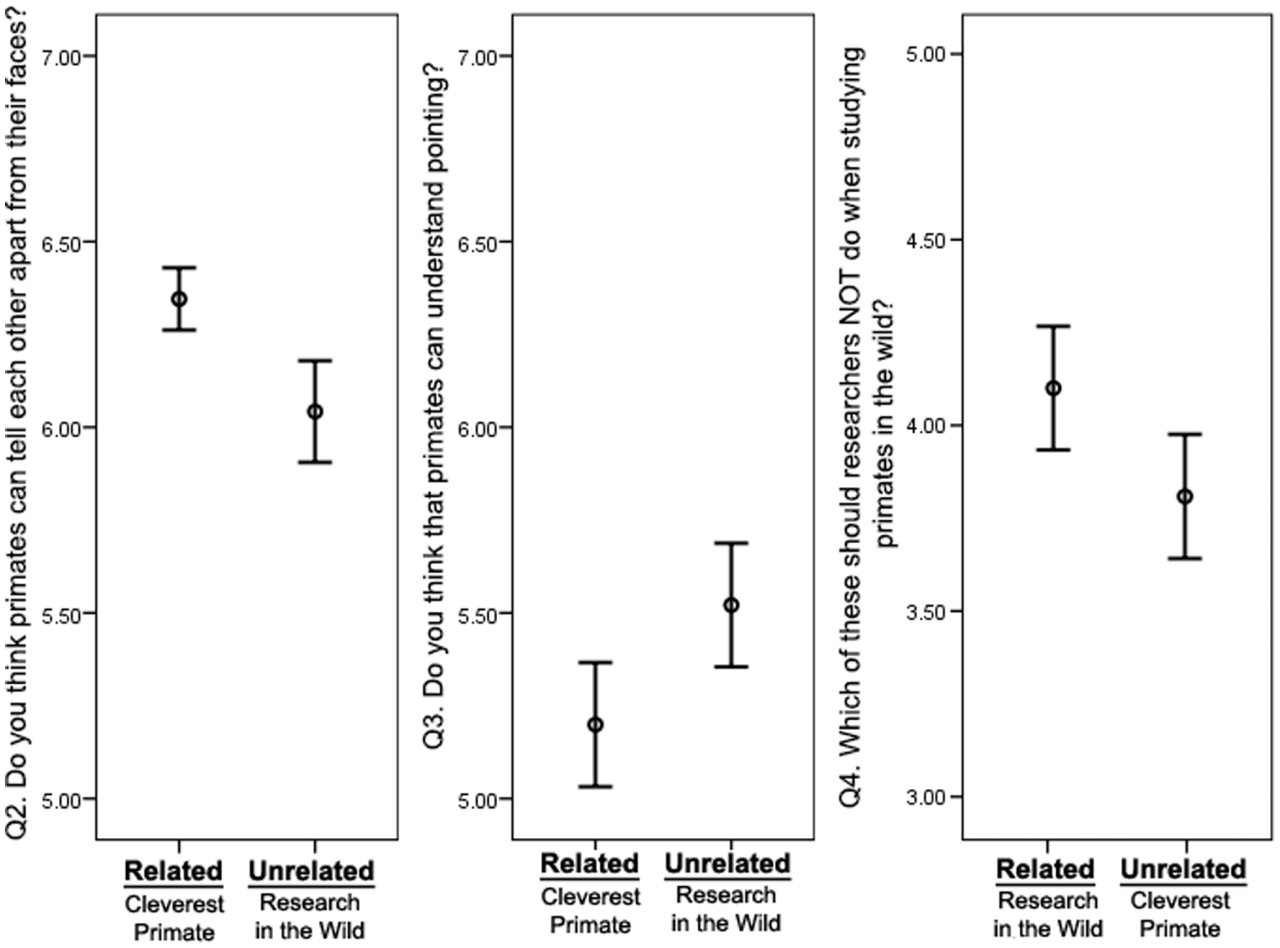
Questionaire responses 2–4. Question responses (mean±SE) of individuals who interacted with the touchscreen. The questions related to topics explained in one game (related game) but not the other (unrelated game). If they had learned correctly, visitors should respond with a higher score in Q2 (primates can tell each other apart from their faces), a lower score in Q3 (primates can't understand pointing), and a higher score in Q4 (that humans should not feed, touch or sneeze near wild animals).

Three of the questions in the questionnaire were directly related to scientific findings/concepts explained in the games. In the ‘Cleverest Primate’ game we demonstrated research showing that facial recognition is not unique to humans, but is instead found in many other primate species and therefore has evolutionary continuity. The ability for non-human primates to recognise and discriminate between faces has been described in many species [14], and if the participant had learnt from this game, they should have had more confidence in the correct answer and in response to Question 2 (‘Do you think that primates can tell each other apart from their faces?’) should have generated a higher score on the scale (1 = Not at all; 7 = Very easily). Participants who played the game containing this information, indeed responded with higher values (more accurately) to this question (Figure 2, Mann Whitney U; Z = −5.18, *p* = <0.001). In the ‘Cleverest Primate’ game we also demonstrated how apes respond to human pointing. Unlike face recognition, pointing is thought to be an ability lacking in non-human primates [15], which we thought could be surprising from a lay perspective. We therefore expected responses to our Question 3 (‘Do you think that primates can understand pointing?’) to be lower (closer to correct answer of 1 = Not at all, compared to 7 = Very easily) if participants had learnt from the game. As predicted, participants who played the ‘Cleverest Primate’ game responded with significantly lower values to this question (Figure 2, Z = −2.24, *p* = 0.025).

In the ‘Research in the Wild’ game, we showed behaviours that would be classified as appropriate or inappropriate for a scientist during field research. Question 4 (‘Which of these should researchers NOT do when studying primates in the wild?’) allowed participants to circle answers from a choice of 5 (3 to be circled, 2 to leave blank). Perfect congruence gave the participant 5 points, 1 incorrect answer gave the participant 4 points, and so on. As expected, participants made more correct choices on this question after playing the ‘Research in the Wild’ game (Z = −2.14, *p* = 0.032; Figure 2).

Participants who reported a higher score (out of 5) on the cleverest primate, also scored higher on question 2 (*rs* = .254, *p* = .002) and question 3 (*rs* = −.177, *p* = .035), congruent with the information in the games. In contrast, participants who reported gaining more badges on the ‘Research in the wild’ game (badges were rewarded for completing each section) showed no difference in score on the field researcher question (*rs* = .197, *p* = .119).

## Discussion

This study demonstrated that implementation of interactive, educational games accessed via touchscreen computers at zoo exhibits can be used for successful public engagement with science and learning of new scientific concepts. Evidence was obtained from quantitative analysis of observational data and questionnaire responses. Two different games were installed, and while both significantly increased dwell times at the exhibit (Marwell) or part of the exhibit (Edinburgh), only one of our games significantly increased interest in science. Despite this, both games increased scientific knowledge and understanding. Therefore, with consideration of appropriate content, conveying information through interactive computerised games seems to be effective.

At the Living Links to Human Evolution Research Centre (also at Edinburgh Zoo), visitors were observed reading the information signage and engaging with interactive devices for around 1 minute per visit [2], and slightly less than this in a study at Lincoln Park Zoo [3]. Although these figures are good, we reported higher average dwell times of 2.5 minutes at the touchscreens. Touchscreen devices, therefore, may be a good accompaniment to static signs and a useful way to communicate scientific information. At Marwell, these figures also implied visitors were often dedicating more than half of their time at the research site to interacting with our touchscreen. Caution must be taken however, as design and positioning of the interactives is also likely to play a big role in how they attract members of the public. It must also be noted, that data collection was taken predominantly outside of the peak times for each site (UK school summer holidays), and these figures could be different when faced with an extremely high visitor flow. As visitor numbers increase, this lowers the proportion of visitors which are likely to play the game (the touchscreen can be monopolized by a single person or group). It is therefore important to note, that although the touchscreen would impact the same amount of people during summer months, this would be a much lower percentage of overall visitors.

Playing the ‘Cleverest Primate’ game increased an interest in science in our participants. This effect is not due to the act of engaging with the device per se, as the second game did not have a similar effect. This effect is likely due to the amount of scientific content which is communicated in the game - of which there was a lot more of in the ‘cleverest primate’ and therefore this could be more impactful on visitor interest in science. Within the ‘Cleverest Primate’ game there are demonstrations of zoo-based primate research, some of the examples coming directly from the research centres at these sites, whereas the ‘Research in the Wild’ game teaches about field research which may seem less relevant to the user at that time and place. It could be that the ‘Cleverest Primate’ game, therefore, afforded a more immersive experience for visitors as it complimented their own observations throughout the exhibits. For the very same reason, this could also explain why visitors tended to report higher levels of enjoyment when playing this game. Finally, it could be that the ‘Cleverest Primate’ game was simply better designed and more fun, and that the counterintuitive nature of many of the scientific findings (e.g. primates don't understand pointing) lead to this increase in interest. The result here is very helpful to future development of interactives, as it will allow us to update our current games (and develop future games) in a way to which will have the biggest impact on public engagement with science; adding more interesting scientific research from wild studies whilst condensing non-scientific content (such as information about living arrangements). Our games did not have an effect on public opinions of conversation issues. It is not surprising however, as each zoo already displays many messages relative to conservation issues and it is likely that visitors had high opinions of these issues prior to playing the game.

The interactive games were specifically targeted at young people (under 16 yrs old) but the games did not have a differential effect on this age group. Instead, the increased interest in science was found in both the younger and older participants (under and over 16 years old), suggesting the benefits from playing the games could be applicable across age groups. However, we did find that younger individuals were significantly more likely to interact with the touchscreens, which implies the impact and reach of our games could be the largest within this sub group. Our second interactive device at Marwell Wildlife (movable flaps containing information underneath regarding macaque facial expressions) attracted all age groups identically, so the touchscreens were particularly attractive to the younger individuals. This study, therefore, provides important evidence that touchscreen devices are an appropriate way to engage young people with scientific information at public exhibits.

Visitors scored better on the knowledge-based questions after they had played the game relevant to the question. This effect was seen from both games, which means even though the ‘Research in the Wild’ game did not significantly increase interest in science in our participants, this game still seemed to facilitate learning. Variability within the game players was also found when looking at their scores on the ‘Cleverest Primate’ game. Individuals who scored higher on the game demonstrated an increased knowledge compared with lower scoring participants. However, this was not seen in the ‘Research in the Wild’ game. Providing competition and rewarding participants for success may play an important role in knowledge retention. Although these results look extremely promising in terms of immediate learning, further research is needed to determine whether knowledge is retained over time as has been demonstrated by other studies [16]. Learning effects have also been demonstrated in response to static information [17] and non-computerised interactive devices [1], but a combination of their appeal to young children and their versatility could make touch-screens a favourable educational tool.

Zoos attract enormous amounts of visitors with a range of demographics (700 million to WAZA zoos per year; http://www.waza.org/en/site/zoos-aquariums), and are engaged in scientific research to a growing extent. Zoos are, therefore, in an excellent position to make a large impact on public understanding of science. Therefore, systematic, quantified analysis of visitor experiences and learning in response to new (and existing) educational devices is extremely important to optimise public engagement with science. Here, we have shown that interactive educational games accessed via computerised touchscreen devices can be an effective method of engagement and learning. The geographically separated study sites allowed us to sample two separate populations in the UK, suggesting it is possible to generalize results to other sites. Further studies should look at the longevity of knowledge gained at research facilities in zoos, and whether education in this environment has long-term impact on educational choices and scientific engagement. Researchers are increasingly accountable for the dissemination of their findings beyond academia, and conducting science in public settings can be an effective method of engaging the public from the very early stages of research.

## Supporting Information

Table S1 **Raw Data.**
(XLSX)
